# Analysis of the conformations of the HIV-1 protease from a large crystallographic data set

**DOI:** 10.1016/j.dib.2017.09.076

**Published:** 2017-10-06

**Authors:** Luigi Leonardo Palese

**Affiliations:** University of Bari "Aldo Moro", Department of Basic Medical Sciences, Neurosciences and Sense Organs (SMBNOS), Bari 70124, Italy

## Abstract

The HIV-1 protease performs essential roles in viral maturation by processing specific cleavage sites in the Gag and Gag-Pol precursor polyproteins to release their mature forms. Here the analysis of a large HIV-1 protease data set (containing 552 dimer structures) are reported. These data are related to article entitled “Conformations of the HIV-1 protease: a crystal structure data set analysis” (Palese, 2017) [1].

**Specifications Table**

**Table:** 

Subject area	*Chemistry, Biology.*
More specific subject area	*Biochemistry, HIV-1 protease structure.*
Type of data	*Table (csv files), text file, figure, animated figures.*
How data was acquired	*Input data for analysis were obtained as pdb files from public database.*
Data format	*Raw: pdb files (as text files). Analyzed: table (csv files), text file, graph, animated GIF.*
Experimental factors	*Raw pdb files were checked for quality.*
Experimental features	*The pdb files included in the database were analyzed by different computational protocols.*
Data source location	*Not applicable.*
Data accessibility	*Analyzed data are within this article.*

**Value of the data**

• The described data set includes a very large number of the public available structures of the HIV-1 protease.
• The database can be useful in the drug design and analysis studies.
• The evidence that preferential conformations are adopted by different sequences could represent an interesting benchmark for the computational prediction and fine tuning of protein structures.

## 1 Data

### 1.1 Data sets

The large HIV-1 protease data set used in the analysis is reported in csv format (file name HIV-1_dataset.csv). Data in this file are arranged in columns (headers in the first row): the first column reports the PDB id of each entry; the second column refers to the internal sequence id; the last two columns report the calculated first and second principal component projections, respectively (calculated by the truncated SVD method [1]). The high quality structures are listed in the file HIV-1_HQ_dataset.csv. In the file are reported the PDB id, the available quality data (R observed, R all, R work, R free, refinement resolution, and the R difference); last column reports the sequence cluster id.

The full set of fluctuations (see [1]) is reported in the file fluctuations.csv. Each row in this file represents an eigenvector (297 eigenvector describe the monomer), and each amino acid is reported as a column (99 amino acid compose the monomer).

The first and second principal modes calculated for the monomer data set are reported as animated GIF image (see [1] for details). Some relevant modes are reported as nmd file [1], [2], [3].

Supplementary material related to this article can be found online at: doi:10.1016/j.dib.2017.09.076.

The following is the Supplementary material related to this article Video 1, Video 2.

Video 1 Mode-1

Video 2 Mode-2

**Fig. 1:**
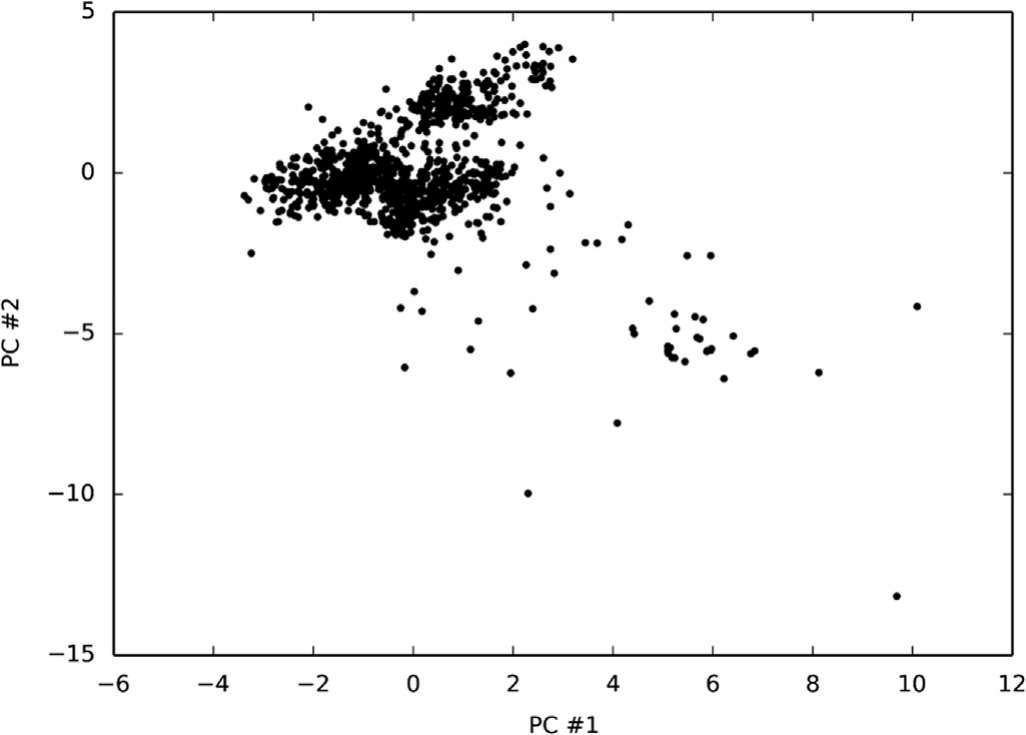
The PCA of the monomer structures calculated by the covariance matrix method.

**Fig. 2:**
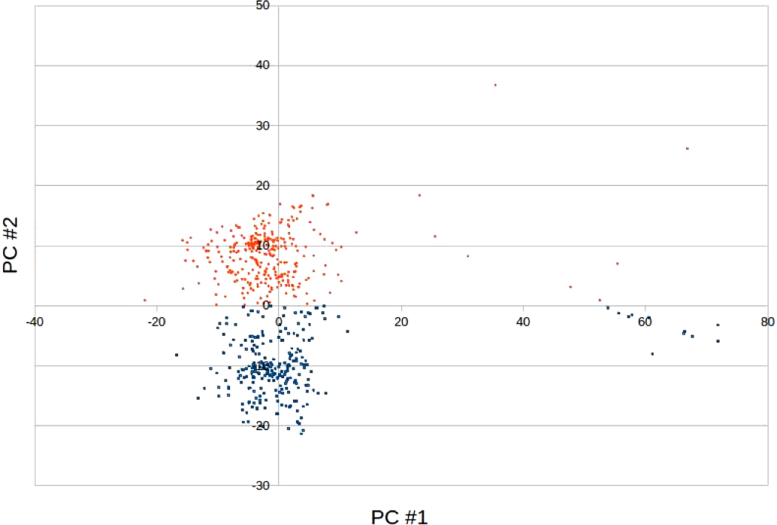
PCA projection of the dimer data set. The entries are colored in blue if their second PC was negative, in red if positive.

**Fig. 3:**
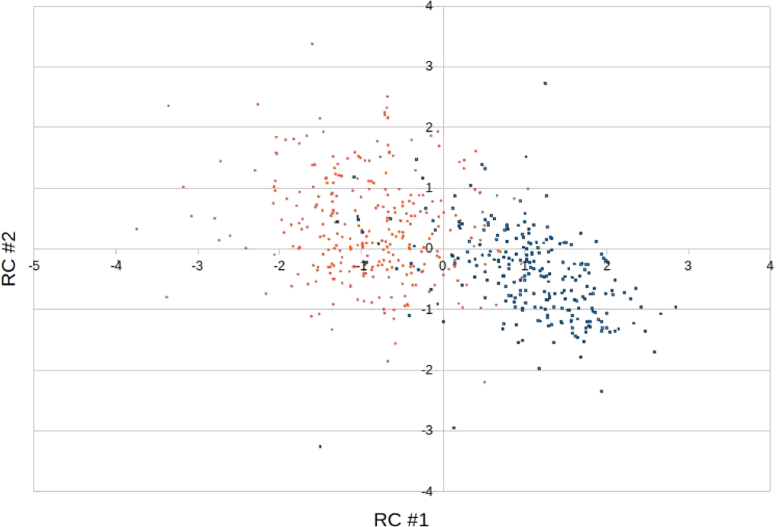
Random projection of the dimer data set. Color code for each entry is the same as in Fig. 2.

**Fig. 4:**
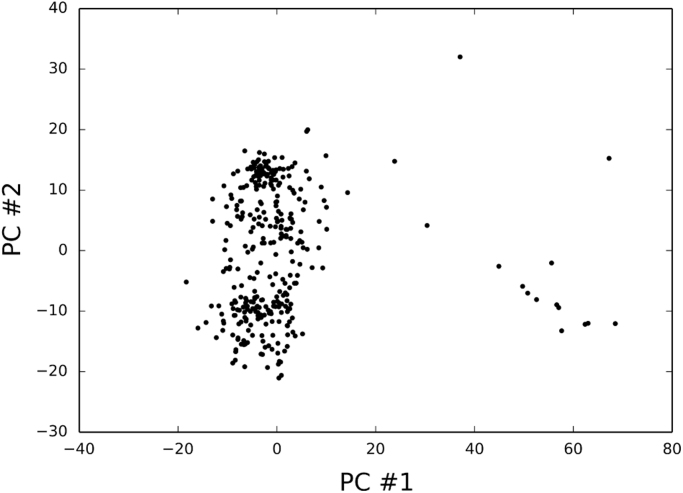
PCA of the HQ dimer data set (truncated SVD method).

Some results of the analysis reported in [1] on the above described data set are reported as Fig. 1, Fig. 2, Fig. 3, Fig. 4. The reader could refers to [1] for full details.

## 2 Relevant sequence clusters in the data set

**Fig. 5:**
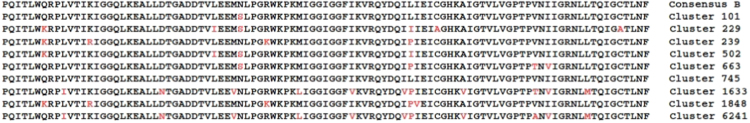
Some sequence groups of the HIV-1 protease data set (see [1]).

Some of the sequence clusters of the HIV-1 protease data set discussed in [1] are reported in Fig. 5; differences respect to the Consensus B sequence (Stanford HIV database) [2], [3], [4], [5] are in red.

## 3 Experimental design, materials and methods

The structures sharing the 90% identity with the Consensus B sequence (Stanford HIV database) [4], [5], [6], [7] were initially considered. The X-ray structures of the HIV-1 protease were obtained from the PDB [8], [9], [10]. A total number of 581 structures in the PDB met this criterion. The structures obtained by X-ray, of dimeric form, classified with an E.C. number 3.4.23.16 (HIV-1 retropepsin), and with a refinement resolution better of at least 3.1 Å were further selected. The number of alpha-carbon atoms in the downloaded pdb files was checked by the bash *grep* function after deleting the multiple conformations by the bash *sed* command. Few structures requested a further manual editing step. Finally 552 HIV-1 protease structures, as dimer, were included in the data set.

The structures contained in a data set were aligned to a common reference by Tcl (www.tcl.tk) scripting in VMD [3]. The new atomic coordinates were stored in a pdb file. For the analysis, the Cartesian coordinates of alpha-carbon atoms of the superposed structures of the data set were extracted and arranged in a matrix form by a Tcl script in VMD. Bracket in the obtained text file were removed in vi (www.vim.org). The result was that the coarse grained data conformations were arranged in a matrix such that each row represented a sample, and each column a degree of freedom. This data matrix was analyzed by methods described in [11], [12], [13], [14], [15], [16], [17], [18], [19], [20], [21], [22], [23], [24], [25], as reported in [1].

## References

[1] Palese L.L. (2017). Conformations of the HIV-1 protease: a crystal structure data set analysis, Biochim.. Biophys. Acta,.

[2] Balkan A., Meireles L.M., Bahar I. (2011). ProDy: protein dynamics inferred from theory and experiments. Bioinformatics.

[3] Humphrey W., Dalke A., Schulten K. (1996). VMD: visual molecular dynamics. J. Mol. Graphics.

[4] Rhee S.-Y., Gonzales M.J., Kantor R., Betts B.J., Ravela J., Shafer R.W. (2003). Human immunodeficiency virus reverse transcriptase and protease sequence database. Nucleic Acids Res..

[5] Shafer R.W. (2006). Rationale and uses of a public HIV drug-resistance database. J. Infect. Dis..

[6] Rhee S.-Y., Kantor R., Katzenstein D.A., Camacho R., Morris L., Sirivichayakul S., Jorgensen L., Brigido L.F., Schapiro J.M., Shafer R.W. (2006). International Non Subtype B HIV-1 Working Group, HIV-1 pol mutation frequency by subtype and treatment experience: extension of the HIVseq program to seven non-B subtypes. AIDS.

[7] Shafer R.W., Jung D.R., Betts B.J. (2000). Human immunodeficiency virus type 1 reverse transcriptase and protease mutation search engine for queries. Nat. Med..

[8] Berman H.M., Westbrook J., Feng Z., Gilliland G., Bhat T.N., Weissig H., Shindyalov I.N., Bourne P.E. (2000). The protein data bank. Nucl. Acids Res..

[9] Berman H., Henrick K., Nakamura H. (2003). Announcing the worldwide protein data bank. Nat. Struct. Biol..

[10] Rose P.W., Prlić A., Altunkaya A., Bi C., Bradley A.R., Christie C.H., Di Costanzo L., Duarte J.M., Dutta S., Feng Z. (2017). The RCSB protein data bank: integrative view of protein, gene and 3D structural information. Nucleic Acids Res..

[11] Raschka S. (2015). Python Machine Learning.

[12] Pedregosa F., Varoquaux G., Gramfort A., Michel V., Thirion B., Grisel O., Blondel M., Prettenhofer P., Weiss R., Dubourg V., Vanderplas J., Passos A., Cournapeau D., Brucher M., Perrot M., Duchesnay E. (2011). Scikit-learn: machine learning in Python. J. Mach. Learn. Res..

[13] Halko N., Martinsson P.-G., Tropp J.A. (2011). Finding structure with randomness: probabilistic algorithms for constructing approximate matrix decompositions. SIAM Rev..

[14] Bro R., Smilde A.K. (2014). Principal component analysis. Anal. Methods.

[15] Bossis F., Palese L.L. (2013). Amyloid beta (1–42) in aqueous environments: effects of ionic strength and E22Q (Dutch) mutation. Biochim. Biophys. Acta.

[16] Palese L.L. (2015). Random matrix theory in molecular dynamics analysis. Biophys. Chem..

[17] Palese L.L. (2015). Correlation analysis of Trp-cage dynamics in folded and unfolded states. J. Phys. Chem. B.

[18] J. Shlens, A Tutorial on Principal Component analysis, arXiv preprint arXiv:1404.1100, 2014.

[19] Van Der Walt S., Colbert S.C., Varoquaux G. (2011). The NumPy array: a structure for efficient numerical computation. Comput. Sci. Eng..

[20] Oliphant T.E. (2007). Python for scientific computing. Comput. Sci. Eng..

[21] L.L. Palese, A Random Version of Principal Component Analysis in Data Clustering, arXiv preprint arXiv:1610.08664, 2016. 10.1016/j.compbiolchem.2018.01.009 29428276

[22] Pérez F., Granger B.E. (2007). IPython: a system for interactive scientific computing. Comput. Sci. Eng..

[23] Hunter J.D. (2007). Matplotlib: a 2D graphics environment. Comput. Sci. Eng..

[24] Palese L.L. (2013). Protein dynamics: complex by itself. Complexity.

[25] Bossis F., Palese L.L. (2011). Molecular dynamics in cytochrome c oxidase Mössbauer spectra deconvolution. Biochem. Biophys. Res. Commun..

