# Supplementary figures and images for: Analysis of the conformations of the HIV-1 protease from a large crystallographic data set
Source: Data Brief. 2017 Oct 6;15:696–700. doi: 10.1016/j.dib.2017.09.076 (PMC5671413; doi:10.1016/j.dib.2017.09.076)

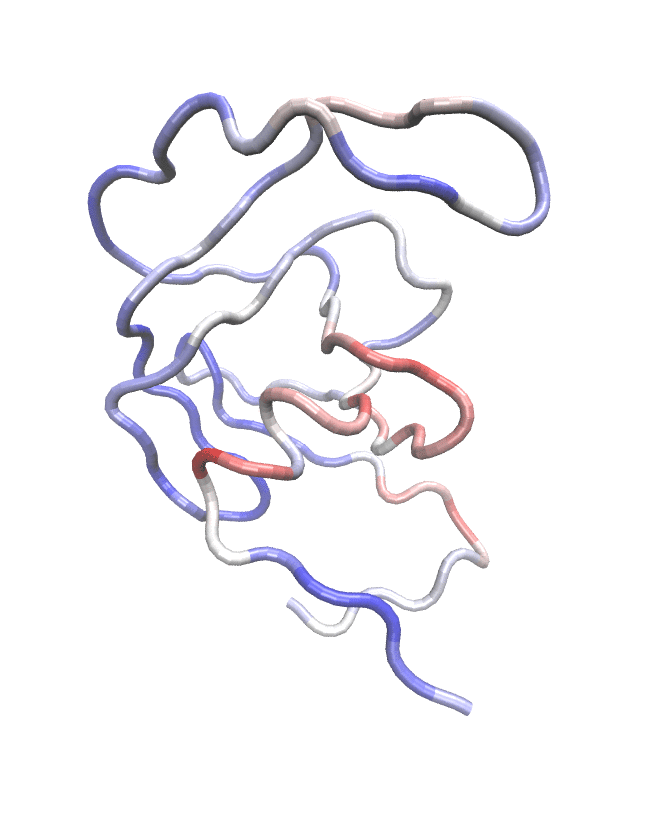

Supplement: Video 1 — Mode-1 [file mmc4.gif]

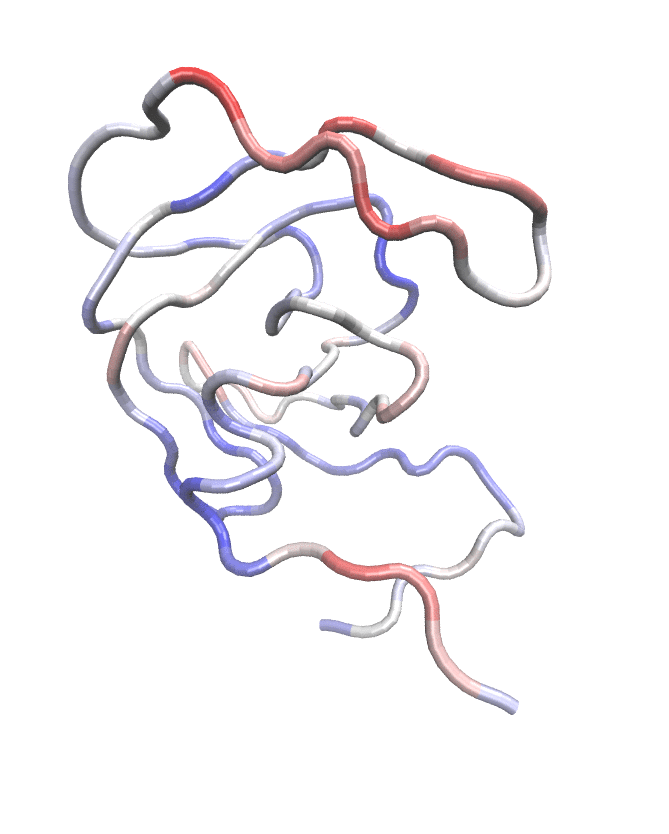

Supplement: Video 2 — Mode-2 [file mmc5.gif]
